# The uterine pathological features associated with sentinel lymph node metastasis in endometrial carcinomas

**DOI:** 10.1371/journal.pone.0242772

**Published:** 2020-11-24

**Authors:** Yuna Kang, Teresa H. Kim, David W. Gjertson, Joshua G. Cohen, Sanaz Memarzadeh, Neda A. Moatamed

**Affiliations:** 1 Department of Pathology and Laboratory Medicine, David Geffen School of Medicine at UCLA, Los Angeles, California, United States of America; 2 Department of Biostatistics, Fielding School of Public Health at UCLA, Los Angeles, California, United States of America; 3 Department of Obstetrics and Gynecology, David Geffen School of Medicine at UCLA, Los Angeles, California, United States of America; University of Campinas, BRAZIL

## Abstract

**Background:**

In recent years, sentinel lymph node excision and ultrastaging have been performed in endometrial carcinomas to obtain information about lymph node status, avoiding unnecessary complete pelvic and paraaortic lymphadenectomy. The purpose of this retrospective study was to provide a comprehensive evaluation of the pathological features of endometrial carcinomas and their significance in association with sentinel lymph node involvement.

**Methods:**

Patients with endometrial carcinomas, preceded by sentinel lymph node mapping, were classified into Group-I and Group-II with negative and positive involvement, respectively. The pathological features, associated with sentinel lymph node involvement, were statistically analyzed, including determination of test performance parameters.

**Results:**

Among 70 patients who had undergone hysterectomy and sentinel lymph node excision, 61 had carcinoma and 9 had atypical hyperplasia. There were 50 patients in Group-I and 10 in Group-II. In Group-II, the significant pathological features were: **1**) lower uterine segment involvement (100%), **2**) an average tumor size of ≥5 CM, **3**) lymphovascular invasion (50%), **4**) cervical stromal invasion (40%), and **5**) depth of myometrial invasion of ≥50% (50%). The incidences of these pathological features were significantly less in Group-I. Statistical analyses singled out “lower uterine segment involvement” as the most important feature.

**Conclusions:**

We have identified five pathological features which are associated with sentinel lymph node involvement. Since lower uterine segment involvement has occurred in all cases of the Group-II cohort, we recommend FIGO and other organizations that determine staging rules should consider whether tumors that involve the lower uterine segment should be staged as higher than “1a”, if the findings in this small series are confirmed by other studies. The results of this study may guide pathologists and oncologists in the diagnostic and therapeutic approaches to management of endometrial carcinomas.

## Introduction

Lymph node status has been an important determinant of prognosis and postoperative treatment planning of endometrial carcinomas since the Federation of International Gynecology and Obstetrics (FIGO) implemented surgical staging in 1988 [1–3]. Since the development of sentinel lymph node (SLN) assessment for melanoma [4, 5], this method has been adopted for surgical staging in a growing number of various other tumors. In recent years, sentinel lymph node assessment has been increasingly performed in endometrial carcinomas as a more targeted approach to obtain information about the lymph node status, avoiding unnecessary complete pelvic and aortic lymphadenectomy, therefore minimizing risks [6, 7]. Sentinel lymph node mapping with ultrastaging has been shown to increase the detection of lymph node metastasis, including micrometastasis, with low rates of false-negative results in patients with uterine-confined diseases [8, 9].

The current recommendation by the National Comprehensive Cancer Network limits SLN sampling to uterine-confined endometrial carcinomas [8]. However, institution-specific approaches may vary, and SLN sampling may be performed for all endometrial carcinomas and even for cases of atypical complex hyperplasia [10].

The pathological features of endometrial carcinomas, in relation to sentinel lymph nodes, have not been fully stratified in the current literature. Therefore, we undertook this retrospective study to evaluate the correlations between specific uterine pathological features and SLN involvement by the disease and reflect on our institutional experience.

## Materials and methods

### Patient selection

This study was reviewed and approved by the Institutional Review Board at David Geffen School of Medicine at UCLA (IRB# 19–000448). Informed consent waivers were obtained because the data were protected and analyzed anonymously. This consecutive retrospective study was carried out by obtaining data through a computer search of our departmental database (by Epic Beaker, Atlanta, Georgia) which included a list of the patients who underwent sentinel lymph node (SLN) dissection and hysterectomy from April 9, 2016 to December 18, 2019.

### Clinical indications for sentinel lymph nodes dissection

In 2014, the National Comprehensive Cancer Network (NCCN) accepted SLN mapping as an alternative to complete lymphadenectomy in apparent uterine-confined endometrial cancers [11]. Prior to hysterectomy, the sentinel lymph node identification was performed by intracervical injection of a tracer for mapping purposes [9].

### Sentinel lymph nodes specimen processing

**Fig 1:**
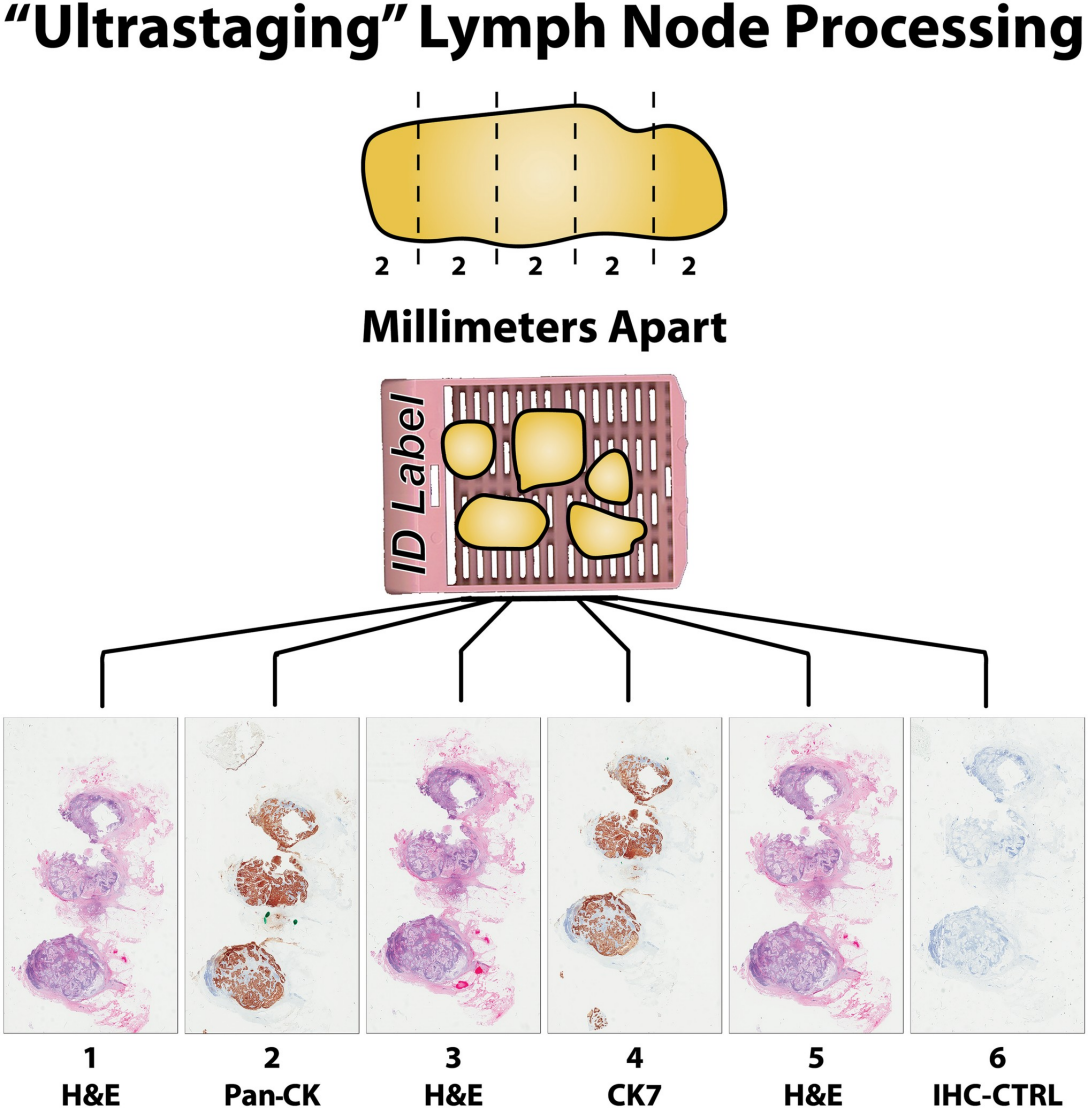
Ultrastaging. This diagram depicts the processing of the resected sentinel lymph node. Each lymph node was cut in 2 mm thick slices and placed in a dedicated tissue cassette and labeled accordingly. Six serial microtome sections were made from each tissue block. Three of them were stained with hematoxylin & eosin (H&E). Serial sections #2 and #4 were stained by immunohistochemistry (IHC) for pancytokeratin (Pan-CK) and cytokeratin-7 (CK7) respectively. CK7 was used to ensure the cancer was not of gastrointestinal origin. Serial section #6 was used as a negative control for IHC.

The excised lymph nodes, with a few exceptions, were labeled as “sentinel lymph node” by the surgeons and were submitted for diagnosis on permanent sections. A lymph node not labeled as such, was considered as a non-sentinel lymph node in pathology, based on the established standard operating procedure (SOP). The location of the lymph nodes and the designation of “sentinel” are all recorded in S1 Table. The submitted SLNs were serially sectioned in the longest axis at 2 mm intervals and totally submitted in the cassettes specified for uterus ultrastaging, according to our protocol for processing uterus-related sentinel lymph node procedures. All of the lymph nodes for “ultrastaging” had three serial sections for hematoxylin and eosin (H&E) stain (serial #1, 3, 5) and three (serial #2, 4, and 6) for immunohistochemistry (IHC). IHC staining included pancytokeratin (AE1/AE3) on serial #2, cytokeratin-7 on serial section #4, and negative control on the serial section#6 (Fig 1). The H&E and IHC procedures were routinely carried out according to the established protocols in our department. No frozen sections were performed on the SLNs and specifically none were performed on the lymph nodes with micro‐metastases or isolated tumor cells.

### Hysterectomy specimen processing

The uteri were processed intraoperatively according to the SOP in our department. After measuring the size and weight, the serosa was inked, and the uteri were cut along the lateral sides. The endometrial tumors were measured and then the endomyometrium was serially sectioned to evaluate the depth of myometrial invasion. A section with the maximum depth of invasion by the tumor, with full thickness of the endomyometrium, and intact serosa, was submitted for frozen sectioning to provide intraoperative evaluation. The type of and grade of the tumors were determined, and the depth of invasion into the myometrium was reported as either <50% or ≥50%. Additional tissue blocks were also submitted for further examination. Tumor diagnoses were made according the established terminologies and FIGO grading [12]. The pathological tumor (T) stage was determined according to the AJCC cancer staging. T-stage “1a” designates tumors limited to the endometrium or invading less than half (<50%) of the myometrium. Stage “1b” is when tumors invade one-half or more (≥50%) of the myometrium. T-stage “2” is referred to as tumors invading the stromal connective tissue of the cervix but still not extending beyond the uterus. T-stage “3” designates tumors involving serosa, adnexa, vagina, or parametrium. T-stage “3a” is when tumors involve the serosa and/or adnexa (direct extension or metastasis) and “3b” designates tumors with vaginal (direct extension or metastasis) or parametrial involvements. T-stage “4” refers to tumors invading the bladder mucosa and/or bowel mucosa [13]. The status of the lymph node’s involvement was included along with the cancer staging in the final pathology reports. All patients with stages other than “1a” were collectively labeled as “>1a” in this study.

### IHC and molecular studies for microsatellite instability

To detect microsatellite instability (MSI), IHC staining of the tumor tissues was performed to evaluate the status of the expression of the mismatch repair (MMR) gene proteins. The absence of reaction was interpreted as “MSI detected.” MSI antibodies were against MutL homolog 1 (MLH1), MutS protein homolog 2 (MSH2), MutS protein homolog 6 (MSH6), and mismatch repair endonuclease (PMS2). In cases where the IHC was interpreted as absent (no nuclear staining), molecular testing of MLH-hypermethylation was ordered for MLH1 and PMS2 to confirm tumor microsatellite instability (T-MSI). If there was a loss of other MMR proteins (MSH2 & MSH6), genetic counseling was recommended for consideration of germline mutation testing to determine if germline microsatellite instability (G-MSI) existed [14, 15]. In either case, any loss of IHC reaction was considered as detected T-MSI.

### Statistical methodologies

To obviate the normality assumptions, Fisher Exact and Wilcoxon’s rank-sum tests were employed to assess associations among the pathological features in cases with SLN involvement versus non-involvement. A *P*-value of 0.05 or less was used to reject the null hypothesis, indicating that a significant difference between the two sets of data existed. Since this work was an observational study where the number of subjects were determined by the available data, no formal power calculations were carried out and all eligible subjects were selected during the defined time-period. Microsoft Excel and Stata statistical software were used to tabulate the data and perform the statistical analyses [16]. Test performance parameters including sensitivity, specificity, positive predictive value (PPV), negative predictive value (NPV), and diagnostic accuracy (DA) were determined according to established methodology [17]. To further observe significant differences, the receiver operating characteristic (ROC) area was calculated by plotting specificity and sensitivity on the X- and Y-axes or simply by dividing the sum of sensitivity and specificity by 2. A perfect diagnostic test has a ROC area of 1.0 while an area of 0.5 denotes a non-discriminating test [17]. To mitigate for spurious significance due to multiple comparisons among pathological features, the Bonferroni’s method was used to calculate and adjust *P*-values as appropriate [18]. In addition, logistic multiple regression analysis was carried out to see if any combination of features might better predict SLN involvement.

### Study design

The cases were classified into two groups: ***Group-I*** (cases with no metastatic) and ***Group-II*** (cases with metastatic) carcinomas in the sentinel lymph nodes. The SLN involvement patterns included isolated tumor cells (ITC), micro-metastatic (Mi) cancer, or macro-metastasis (M). The SLNs with ITC were lymph nodes that contained single or barely visible clusters (<0.2 mm) of tumor cells on H&E sections. Cytokeratin IHC staining was required to identify or confirm ITC. Micro-metastases were clusters (≥0.2 mm but ≤2.0 mm) of tumor cells on H&E slides which may have required the IHC staining for confirmation. Macro-metastases were clusters (>2.0 mm) of tumors which may not have required the cytokeratin stain. These definitions were adopted from the breast cancer classifications by the American Joint Committee on Cancer (AJCC) and the College of American Pathologists (CAP) [13]. The presurgical biopsy as well as the postsurgical hysterectomy diagnoses were tabulated (S1 Table). The hysterectomy diagnoses were “complex atypical hyperplasia,” “endometrioid adenocarcinoma,” “serous carcinoma” and “no residual tumor,” which were tabulated accordingly (S1 Table). In the tabulations, the histological grade, lower uterine segment involvement (LUSI), cervical stromal involvement (CSI), lymphovascular invasion (LVI), tumor size (greatest dimension in centimeters), depth of myometrial invasion (<50% versus ≥50%), pathological stage, and MSI status were recorded. To avoid frozen artifacts clouding our diagnostic interpretation, the presence or absence of LVI was assessed on permanent sections of the tissues which were not used during the intraoperative consultation. Depth of invasion (DI) was measured on frozen sections (if performed) and permanent sections (S1 Table). The greatest measurement was used to determine pathological stage. These features were statistically compared between the two groups. For ease of cross referencing, when extracting ***Group-I*** and ***Group-II*** from the S1 Table, the same sequential case numbers were preserved throughout their respective tables. Each group had 2 arms, one was combined endometrioid and serous carcinomas (ECA+SCA) and the other only included endometrioid carcinomas (ECA). Statistical analyses were carried out for the two arms in each group.

## Results

### Overall

**Fig 2:**
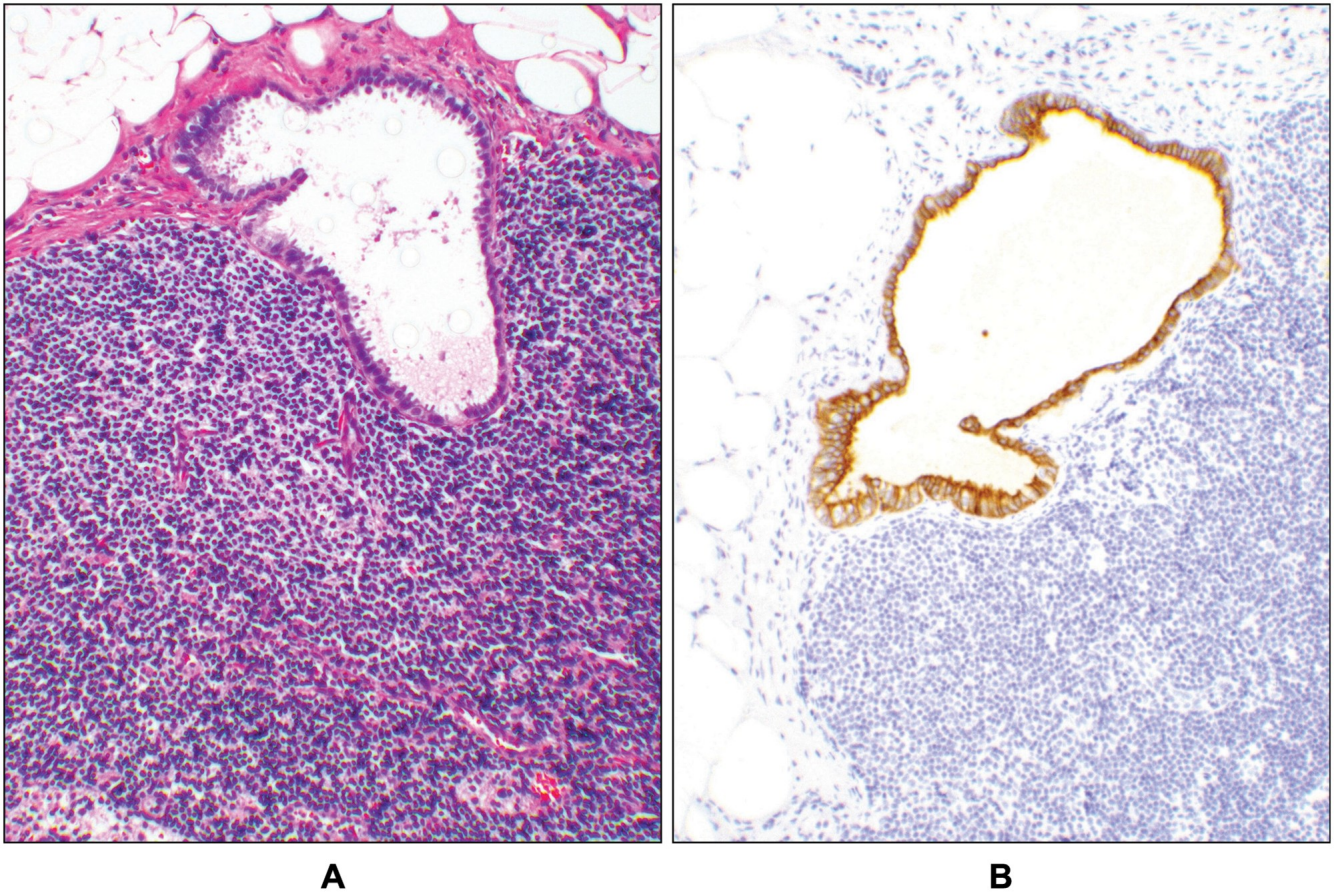
Endosalpingiosis. A negative sentinel lymph node with endosalpingiosis in a patient with complex atypical hyperplasia (case # 6, S1 Table). (**A**) The photomicrograph shows a cystic glandular structure in the lymph node that is lined with fallopian tube-type ciliated epithelium on hematoxylin and eosin (H&E) stain. (**B**) Immunohistochemical stain for cytokeratin highlights the glandular structure at the same location as on the H&E slide (10x objective).

During the period of March 2016 to April of 2019, a total of 70 cases of robotic laparoscopic hysterectomies with sentinel lymph node mapping were identified at our medical center. The patients’ ages ranged from 33–85 years with a median age of 62. Of these cases, 12 patients were diagnosed as having complex atypical hyperplasia (CAH) on the initial biopsy. Based on the findings in the hysterectomy specimens, the diagnosis of CAH was confirmed in 7 cases. The other 5 cases were upgraded to carcinoma after review of the hysterectomy specimens. Subsequently, the cases with confirmed diagnoses of CAH were excluded from the two groups below (S1 Table). One of the 7 patients with CAH had a MSH6 germline mutation (case #5, S1 Table). In 2 cases, although the diagnosis was ECA on the initial biopsy, no residual tumor was identified in the hysterectomy specimens (cases #62 & 63, S1 Table); these cases were also excluded from the two groups. The pathological features as well as the tumor stages of all specimens were summarized in S2 Table. Among the cases with negative sentinel lymph nodes, 3 had endosalpingiosis (Fig 2), a benign incidental finding (cases# 6, 35, and 43, S1 Table). Overall, 81.5% (57 cases) of the cases had successful bilateral sentinel lymph node dissection (S1 Table). Among the remaining 18.5%, the SLNs were identified and resected unilaterally by the surgeon of which 12 were on the left and 1 on the right. Almost all SLN-labeled samples contained multiple lymph nodes per case and the sites are detailed in S1 Table. In addition to SLN dissection, some cases also had non-sentinel lymph nodes removed. These cases were either scheduled to have addition non-SLN dissections prior to the surgery or intraoperative findings led the surgeons to perform the dissection. In one case (case# 70, S1 Table), the specimen labeled as “SLN”, had only fibrofatty tissue, therefore, the surgeons resorted to bilateral non-SLN lymphadenectomy where metastasis was discovered in one paraaortic lymph node on the right side while the left pelvic SLN was negative. Subsequently, this case was excluded from the study groups.

### Group-I

Group-I included 50 patients with endometrial carcinomas diagnosed on the hysterectomy specimens and had no lymph node metastases (S3 Table). The success rate of bilateral SLN resection was 82% (41 cases) in this group. The remaining 9 cases had their lymphadenectomy only on the left side. In this group, 44 had ECA and 6 had high grade SCA; ages ranged from 33 to 85 years with a median age of 64. Five cases had a diagnosis of CAH on the initial biopsy but a final diagnosis of ECA on the hysterectomy specimen (cases #8–12, S3 Table). Another patient had an initial diagnosis of SCA which was changed to ECA after hysterectomy (case #61, S1 and S3 Tables). The most common histological grade in this group was grade I (56%). The average tumor size was 2.61 CM with LUSI seen in 16%, CSI in 4%, and LVI in 6% of the cases. Among these patients, 82% had <50% and 18% had ≥50% depth of invasion into the adjacent myometrium. Also, 82% had a T-stage of 1a disease and 18% had T-stages higher than 1a. Tumor MSI was present in 26% of the cases in this group. The significant findings are summarized in S4 Table.

### Group-II

**Fig 3:**
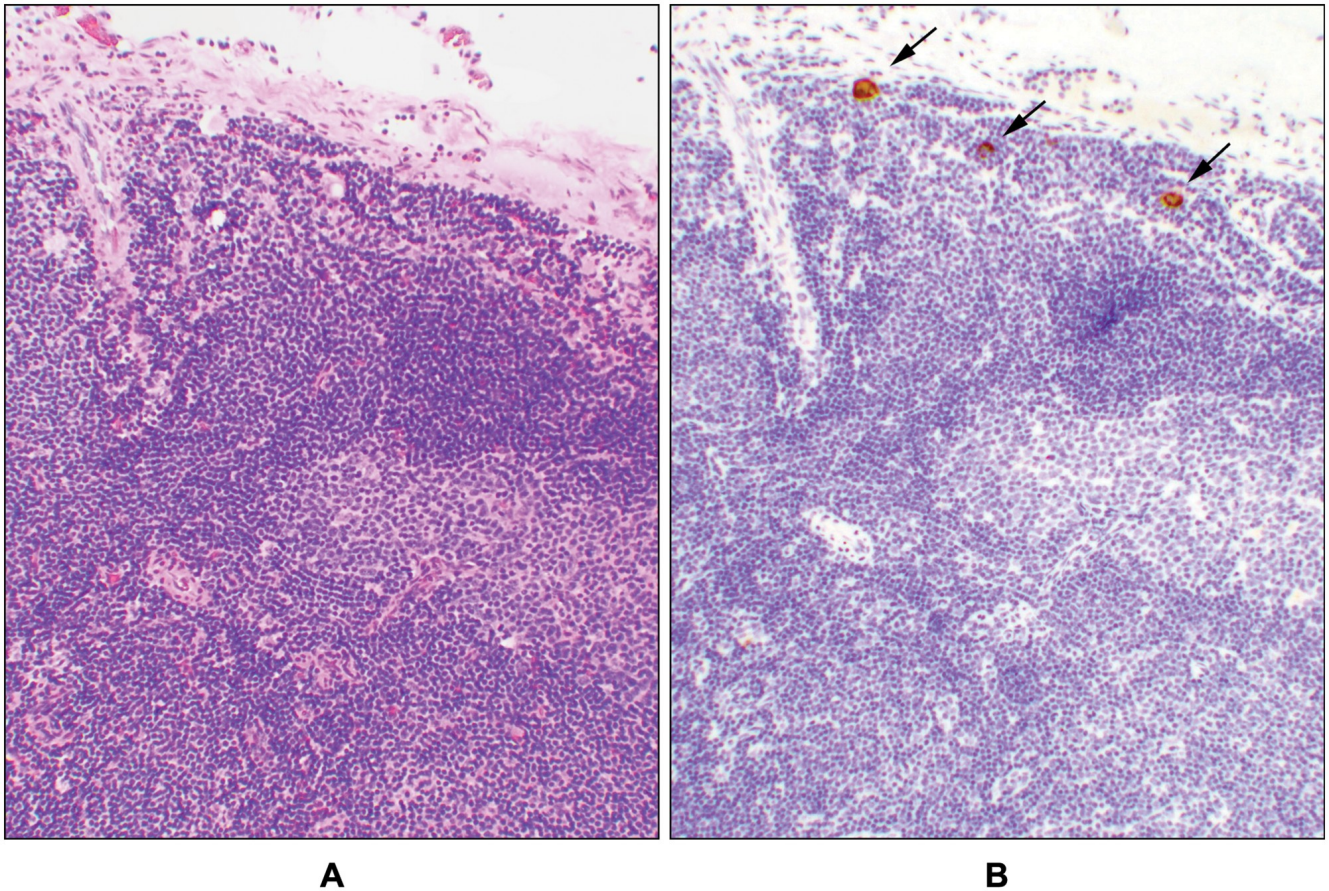
Isolated tumor cells. A sentinel lymph node with isolated tumor cells (case # 50, S1 and S5 Tables). (**A**) The photomicrograph shows a portion of a normal-appearing lymphoid structure with no obvious tumor cells by hematoxylin and eosin (H&E) stain. (**B**) Few isolated tumor cells were highlighted by cytokeratin immunohistochemical stain (arrows) within the same area of the lymph node as seen on the H&E slide (10x objective).

**Fig 4:**
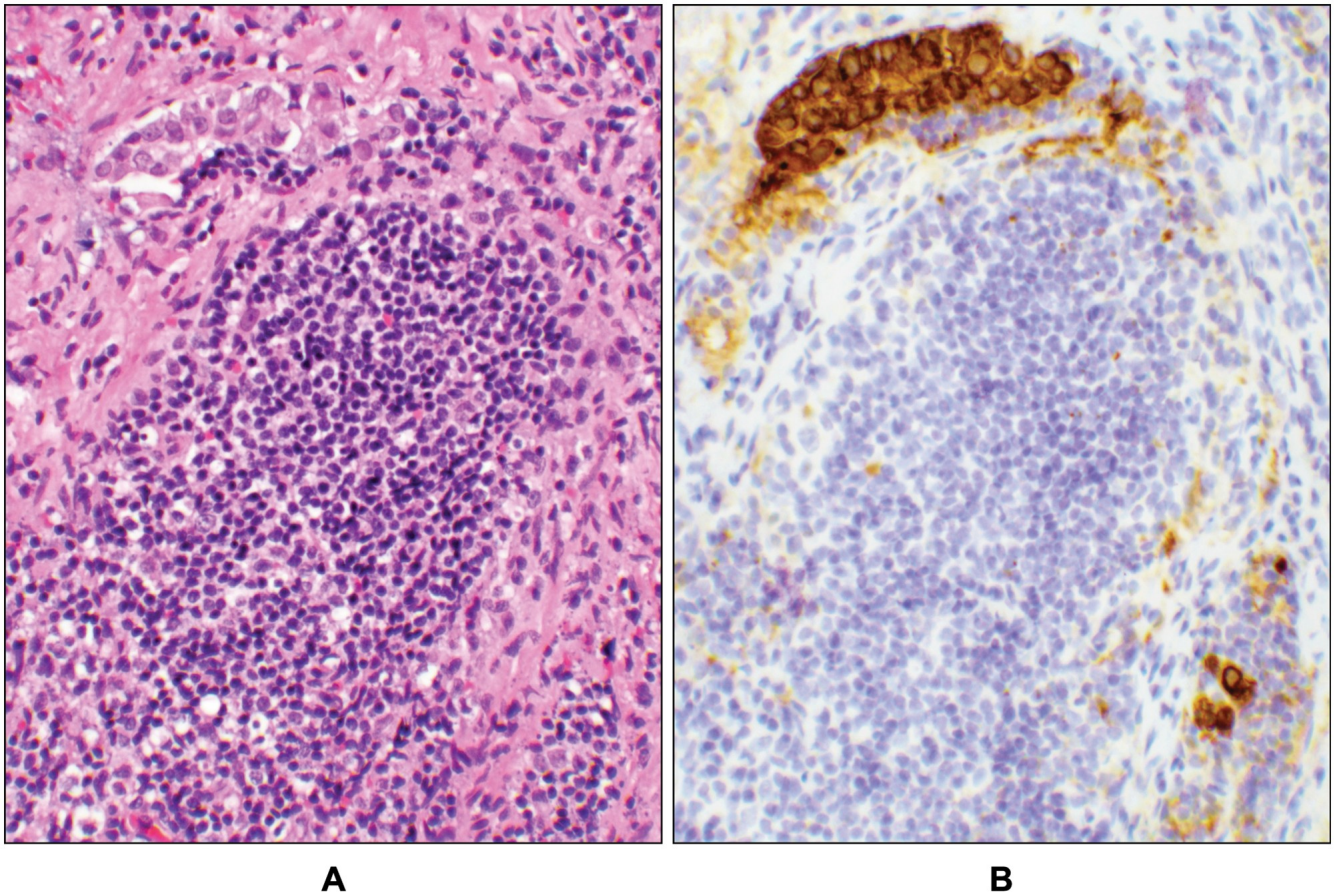
Micrometastasis. A sentinel lymph node with micrometastatic carcinoma (case # 58, S1 and S5 Tables). (**A**) A cluster of tumor cells seen by hematoxylin and eosin (H&E) stain. (**B**) Cytokeratin immunohistochemical stain highlighted the same focus of the tumor seen on the H&E slide (10x objective).

**Fig 5:**
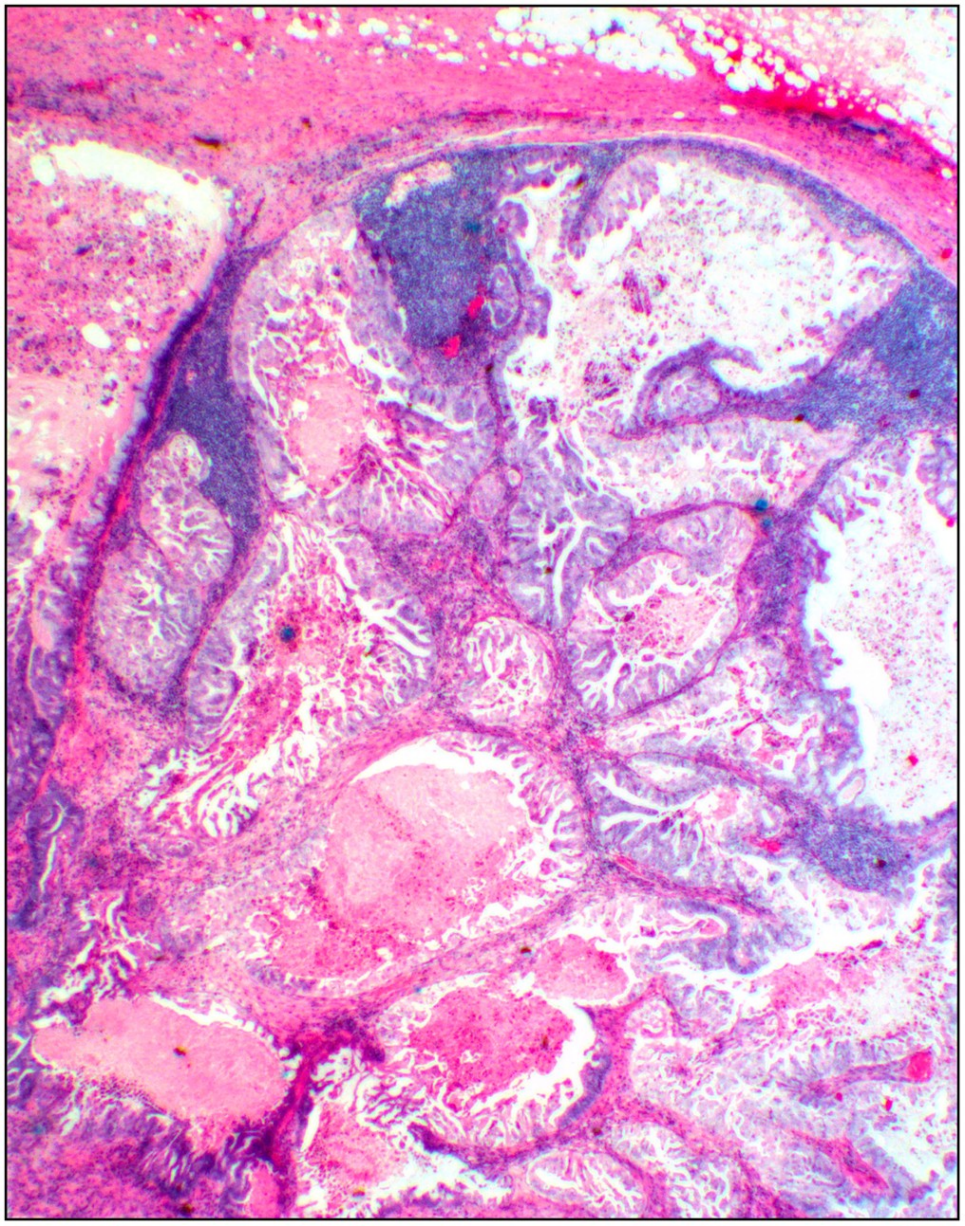
Metastasis. A sentinel lymph node with a metastatic carcinoma (case # 54, S1 and S5 Tables). Hematoxylin and eosin stain shows almost complete effacement of the lymph node by endometrioid adenocarcinoma. Small clusters of remnant lymphoid cells are present (2x objective).

This group included 10 patients with endometrial carcinomas with sentinel lymph node involvement (S5 Table). All 10 cases had ECA; ages ranged from 38 to 79 years with a median of 65 (cases 50–59, S5 Table). The success rate for bilateral SLN resection was 90% in this group (S5 Table). Four cases had isolated tumor cells (Fig 3), two with micro- (Fig 4), and four had macro- (Fig 5) metastases (S5 Table). In these 10 patients, the SLN labeled nodes were pelvic, among which two patients (cases# 56 & 57, S5 Table) had also metastatic carcinoma in their paraaortic non-SLNs. All cases were also evaluated for the presence of adenomyosis involved by the tumor and for the microcystic-elongated-fragmented (MELF) pattern of invasion. Neither of these two features were present.

The most common histological grade in this group was grade 2 (60%). The average size of the tumor was 5.62 CM in the greatest dimension. LUSI was seen in 100%, CSI in 40%, and LVI in 50% of the cases. Five cases (50%) had <50% and five (50%) had ≥50% depth of invasion into the myometrium. In this group, 30% of the patients had T-stage “1a” disease while 70% had stages higher than “1a”. The cases with stages higher than “1a” included three cases with stage “1b”, two with stage “2”, and three with stage “3”. MSI was present in three (30%) of the cases. The findings in this group were also summarized in S4 Table.

### Statistical analyses

Statistical analyses were carried out to compare the features in the context of the negative (Group-I) versus positive SLNs (Group-II). Each of the features is discussed separately below and summarized in Tables 1 and 2.

**Table 1:** Fisher’s Exact comparison of the SLN status versus the tumor features as individually stated in the sub-tables.

Group	ECA & SCA		ECA	
**Table 1a. LUSI** vs **SLN** status				
	**LUSI-NI**	**LUSI-Pr**	**LUSI-NI**	**LUSI-Pr**
**GI** (Neg SLN)	42 (84%)	8 (16%)	37 (84%)	7 (16%)
**GII** (Pos SLN)	0 (0%)	10 (100%)	0 (0%)	10 (100%)
***P*-Value**	**0.0000001**		**0.0000008**	
***(BC*: *x5) P*-Value**	**0.0000006**		**0.000004**	
**Table 1b. CSI** vs **SLN** status				
	**CSI-NI**	**CSI-Pr**	**CSI-NI**	**CSI-Pr**
**GI** (Neg SLN)	48 (96%)	2 (4%)	42 (95%)	2 (5%)
**GII** (Pos SLN)	6 (60%)	4 (40%)	6 (60%)	4 (40%)
***P*-Value**	**0.005**		**0.008**	
***(BC*: *x5) P*-Value**	**0.03**		**0.04**	
**Table 1c. DI** vs **SLN** status				
	**DI: <50%**	**DI: ≥50%**	**DI: <50%**	**DI: ≥50%**
**GI** (Neg SLN)	41 (82%)	9 (18%)	35 (80%)	9 (20%)
**GII** (Pos SLN)	5 (50%)	5 (50%)	5 (50%)	5 (50%)
***P*-Value**	**0.04**		**0.1**	
***(BC*: *x5) P*-Value**	**0.2**		**0.5**	
**Table 1d**. **T-Stage** vs **SLN** status				
	**T-Stage 1a**	**T-Stage >1a**	**T-Stage 1a**	**T-Stage >1a**
**GI** (Neg SLN)	41 (82%)	9 (18%)	35 (80%)	9 (20%)
**GII** (Pos SLN)	3 (30%)	7 (70%)	3 (30%)	7 (70%)
***P*-Value**	**0.002**		**0.004**	
***(BC*: *x5) P*-Value**	**0.01**		**0.02**	
**Table 1e. LVI** vs **SLN** status				
	**LVI-NI**	**LVI-Pr**	**LVI-NI**	**LVI-Pr**
**GI** (Neg SLN)	47 (94%)	3 (6%)	41 (93%)	3 (7%)
**GII** (Pos SLN)	5 (50%)	5 (50%)	5 (50%)	5 (50%)
***P*-Value**	**0.002**		**0.003**	
***(BC*: *x5) P*-Value**	**0.01**		**0.02**	

**ECA**, endometrioid adenocarcinoma; **SCA**, serous carcinoma; **Neg**, negative; **Pos**, positive; **SLN**, sentinel lymph node; **NI**, not identified; **Pr**, present; **LUSI**, lower uterine segment involvement; **CSI**, cervical stromal involvement; **LVI**, lymphovascular invasion; **>1a**, includes 1b, 2, 3, 3a; **DI**, depth of invasion; **T-Stage**, tumor stage; **vs**, versus; **BC**, Bonferroni multiple testing correction of the P-Values of the five pathological features by multiplying each P-Value by 5.

**Table 2:** Wilcoxon Rank-Sum test, comparison of the T-size versus SLN status.

T-Size	ECA + SCA		ECA	
	Neg SLN	Pos SLN	Neg SLN	Pos SLN
**n**	50	10	44	10
**Mean, Diameter (CM)**	2.61	5.62	2.82	5.62
**Std Dev**	1.86	1.76	1.87	1.76
***P*-Value**	**0.0002**		**0.0004**	
***(BC*: *x5) P*-Value**	**0.001**		**0.002**	

**ECA**, endometrioid adenocarcinoma; **SCA**, serous carcinoma; **Neg**, negative; **Pos**, positive; **SLN**, sentinel lymph node; **Std Dev**, standard deviation; **T-Size**, tumor size; **BC**, Bonferroni multiple testing correction of the *p*-values of the five pathological features by multiplying each *p*-value by 5.

### Lower uterine segment involvement

Lower uterine segment involvement was present in only 16% of the patients in Group-I who did not have any lymph node metastasis in contrast to 100% in Group-II (Table 1a). In a 2x2 table, Fisher’s Exact analysis yielded a *P*-value of <0.0001 when the presence of LUSI was examined for the patients with either “ECA & SCA” or “ECA” in Group-I versus Group-II. After Bonferroni’s correction, the *P*-values for LUSI remained significant (Table 1a).

### Cervical stromal involvement

Uterine cervical stromal involvement was seen in 4% of the cases in Group-I, in contrast to 40% of Group-II. The Fisher’s Exact analysis yielded a *P*-value of <0.01 when the presence of CSI was examined in the two groups for a combined ECA+SCA as well as for ECA alone. The *P*-values stayed significant following the multiple testing correction procedure (Table 1b).

### Depth of invasion

Depth of myometrial invasion was first determined grossly on the freshly cut specimen during the intraoperative consultation, then finalized after reviewing the permanent sections. The greatest measurement was used for staging purposes. In Group-I, 18% of the cases had a ≥50% DI in contrast to 50% in Group-II. Fisher’s Exact *P*-value was 0.04 when DIs were compared between the two groups for combined ECA+SCA. However, the *P*-value was 0.1 for only ECAs. The *P*-value became insignificant following Bonferroni’s correction (Table 1c).

### Pathological tumor stage

Pathological T-stage was determined following hysterectomy. Nine patients (18%) in Group-I had “>1a” T stage, while 70% of the cases in Group-II had “>1a” T-stage. In a 2x2 table analysis, the Fisher’s Exact test resulted in a *P*-value of 0.002 when comparing the pathological stages of the two groups. The *P*-values remained significant even after the multiple testing correction process for the five pathological features (Table 1d).

### Lymphovascular invasion

Lymphovascular invasion was histologically observed in 6% of patients in Group-I as opposed to 50% in Group-II. Fisher’s Exact analysis for this feature yielded a *P*-value of <0.01when presence of LVI was examined in both groups for combined ECA+SCA as well as for ECA alone. The *P*-values stayed significant even following Bonferroni’s procedure (Table 1e).

### Tumor size (T-Size)

The average tumor sizes were 2.61 and 5.62 CMs in Group-I and Group-II respectively. Wilcoxon Rank-Sum comparison of the data sets in the two groups resulted in *P*-values of <0.001. After multiple testing corrections, the *P*-values were <0.01 (Table 2).

### Tumor microsatellite instability

There were 17 cases with T-MSI abnormality, 13 (26%) in Group-I, and 3 (30%) in Group-II. The remaining one case was diagnosed with CAH which was excluded from our study groups. All cases with MSI had a diagnosis of ECA except for one who had SCA (in Group-I). In addition to tumor MSI, this patient also had a germline mutation (case# 68, S1 Table). A *p*-vale of 1 was obtained when T-MSI was compared between the two groups. Additionally, when T-MSI was compared with LUSI, the *p*-value was 0.5 (S6 Table).

### Tumor histological grade

The tumor histological grades were compared in 2x2 tables using Fisher’s Exact test. For combined ECA+SCA and ECA alone, all *p*-values were greater than 0.05 (S7 Table). All comparative analyses for the two groups are listed in S7 Table. Since FIGO grades I & II are considered as low grade tumors [19], they were tested together versus FIGO grade III which also yielded a *p*-value of 0.7 (S7 Table).

Based on the findings of the above statistical analyses, five pathological features emerged to be associated with the sentinel lymph node involvement which are *lower uterine segment involvement*, *tumor size*, *lymphovascular invasion*, *cervical stromal invasion*, and *depth of invasion*, in the order of importance (Table 3).

**Table 3:** The pathological features associated with sentinel lymph node involvement in endometrial carcinomas, listed by the importance based on the *p*-values.

Pathology Features	ECA + SCA		ECA	
	P-Value	BC-PV	P-Value	BC-PV
1. **LUSI** (Table 1a)	0.0000001	0.0000006	0.0000008	0.000004
2. **T-Size** (Table 2)	0.0002	0.001	0.0004	0.002
3. **LVI** (Table 1e)	0.002	0.01	0.003	0.02
4. **CSI** (Table 1b)	0.005	0.03	0.008	0.04
5. **DI** (Table 1c)	0.04	0.2	0.1	0.5

**ECA**, endometrioid adenocarcinoma; **SCA**, serous carcinoma; **LUSI**, lower uterine segment involvement; **T-Size**, tumor size of ≥5 CM; **LVI**, lymphovascular invasion; **CSI**, cervical stromal involvement; **DI**, depth of myometrial invasion of ≥50%; **BC**, Bonferroni multiple testing correction of *p*-values of the five pathological features by multiplying each *p*-value by 5; **PV**, *p*-value.

After Bonferroni correction for multiple testing, all *p*-values continued rejecting null hypothesis except for DI where the *p*-value rose to 0.2 for ECA+SCA and 0.5 for ECA (Table 3), respectively.

### Tests performance analyses

Emergence of the significance of the five pathological features (tests) led to their analyses for diagnostic accuracy measures. Since these features occurred prominently in Group-II, we assumed their presence would be considered as “true positive” in Group-II and “false positive” in Group-I. Conversely, their absence would be “true negative” in Group-I and “false negative” in Group-II (S8 Table). Based on these assumptions, the results were summarized in Table 4.

**Table 4:** Test measures of the five pathologic features associated with the sentinel lymph node involvement in endometrial carcinomas.

	LUSI	T-Size	LVI	CSI	DI
True Positive	10	7	5	4	5
True Negative	42	44	47	48	41
False Positive	8	6	3	2	9
False Negative	0	3	5	6	5
**Sensitivity**	100%	70%	50%	40%	50%
**Specificity**	84%	88%	94%	96%	82%
**PPV**	56%	54%	63%	67%	36%
**NPV**	100%	94%	90%	89%	89%
**Diagnostic Accuracy**	87%	85%	87%	87%	77%
**ROC Area**	**0.92**	0.79	0.72	0.68	0.66
**MR ROC Area**	---	**0.90**			

**LUSI**, lower uterine segment involvement; **T-Size**, tumor size of ≥5 CM; **LVI**, lymphovascular invasion; **CSI**, cervical stromal involvement; **DI**, depth of myometrial invasion of ≥50%; **PPV**, positive predictive value; **NPV**, negative predictive value; **ROC**, receiver operating characteristic calculated by (sensitivity + specificity)/2; **MR**, multiple regression for grouping yielded a single ROC for the four features with LUSI excluded.

These findings showed that LUSI had a sensitivity of 100% and the highest ROC area of 0.92 in this series. All five tests had a specificity of 82% or greater while LVI and CSI had the highest values which were 94% and 96%, respectively. Also, all the tests had a diagnostic accuracy of 85% or greater except for DI (77%). For each feature, the test accuracy measures are detailed in Table 4.

Multiple regression analysis singled out LUSI as the single most significant feature associated with SLN involvement for its sensitivity of 100% and absence of the involvement in 42 patients. Therefore, these 42 cases were excluded further from the statistical analysis. The remaining 18 cases were insufficient to judge the association for the other 4 pathological features. Alternatively, if markers are ignored in this multiple regression analysis, then the remaining 4 features performed collectively about as well (ROC = 0.90) as a single feature (Table 4).

## Discussion

**Fig 6:**
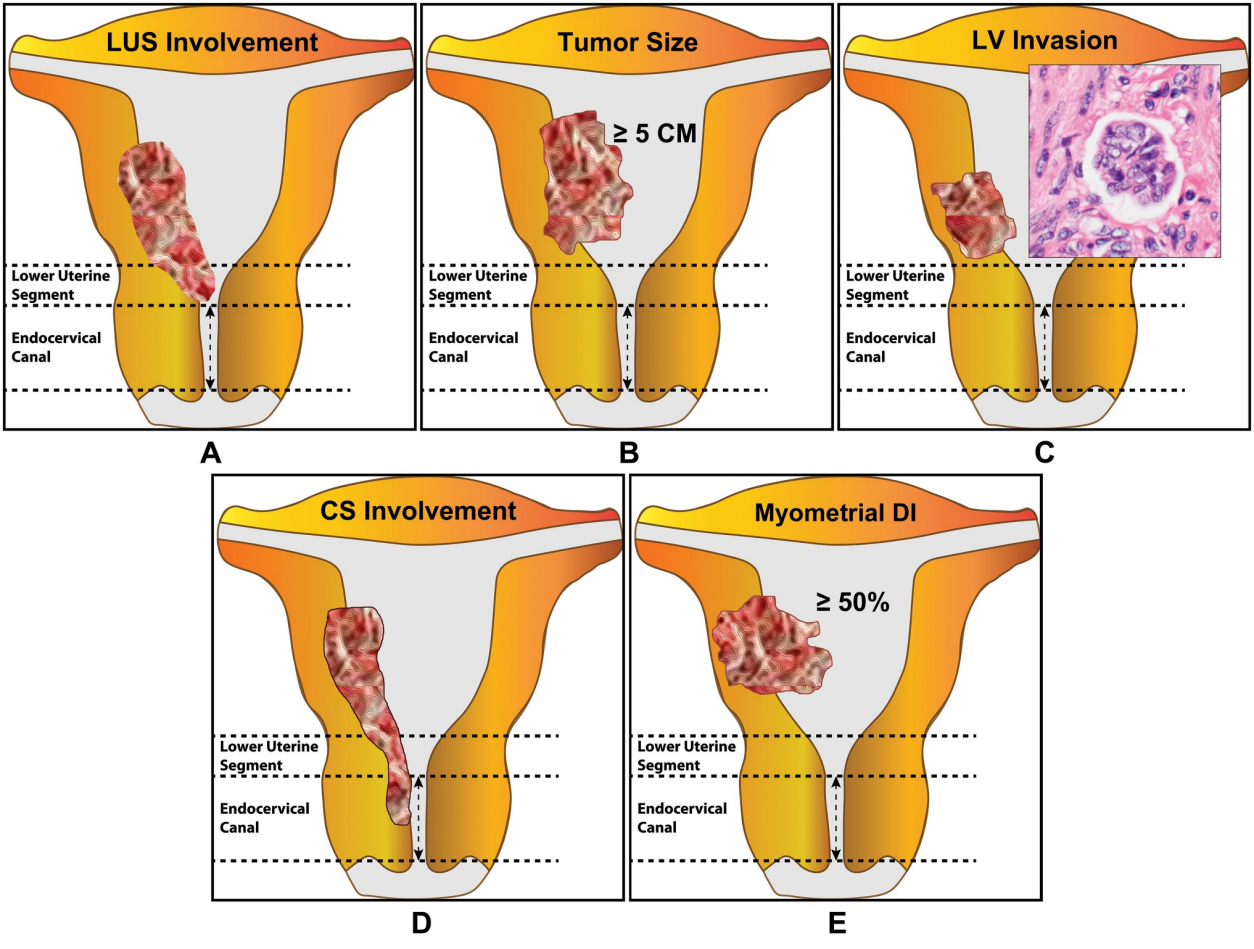
Pathological features associated with sentinel lymph node involvement. There are five uterine pathological features which are associated with sentinel lymph node (SLN) involvement. The figures are arranged according to their importance as listed in Table 3. (**A**) Lower uterine segment (LUS) involvement was present in 100% of the cases with positive SLNs as opposed to <20% in those cases with negative lymph nodes. (**B**) In Group-II, cases with positive SLN had an average tumor size of 5 CM or greater. (**C**) Histological lymphovascular (LV) invasion was observed in 55% of the cases with positive SLNs but 6% in those with negative nodes. (**D**) Cervical stromal (CS) involvement had occurred in 36% of patients with positive SLNs while only in 4% of those who has no SLN metastasis. (**E**) A myometrial depth of invasion (DI) of ≥50% occurred in >50% of the patients with positive SLNs while it was seen in <20% of those with negative SLNs.

The results of this study reaffirm the suitability of sentinel lymph node resection as an instrumental step toward improving prognostication and therapeutic approaches in endometrial carcinomas. The procedure helps to determine whether the endometrial cancer is still locally confined, or if it is on its way to become systemic. Through this study, we have stratified the pathological features of endometrial carcinomas that are highly associated with SLN metastasis. Five pathologic features, if present, appear to be significantly associated with SLN involvement which are outlined in Fig 6. Further, staging of the tumor depends on two of these elements, depth of myometrial invasion (≥50%), and cervical stromal invasion [13, 20]. Stage “1a” represents a tumor that has less than 50% DI and has no CSI, otherwise the cancer will be staged as “1b” or “2” respectively. Any T-stage greater than “1a” is also significantly associated with SLN metastases (Table 1d). Therefore, these pathological factors will be conducive to assess the status of the tumor and the need for further therapeutic management and follow up. For all parameters studied, we looked at ECA tumors alone and ECA combined with SCA (Table 1). The statistical behavior of these two subcategories were similar for all parameters except for depth of invasion (Table 1c) where *p*-values were 0.04 and 0.1 for ECA+SCA and ECA arms, respectively. These variations are most likely due to the small number of SCA cases in this series. In general, uterine serous carcinoma accounts for 10% of uterine cancers which have an aggressive nature with a high mortality rate [21]. Schiavone et al. have likewise suggested performing SLN mapping in SCA instead of routine lymphadenectomy [21]. Kennard et al. demonstrated that patients with a high risk histology (non-endometrioid histology with any degree of myometrial invasion) have more metastases in both SLNs and non-SLNs than low to intermediate risk patients [22]. However, a meta-analysis of SLN assessment has determined that non-endometrioid carcinomas (serous and clear cell types) were not associated with any significant differences in SLN detection compared with endometrioid carcinomas [23]. A larger cohort of patients with SCA would shed more light on the prognosis of these patients undergoing SLN resection.

NCCN guidelines denote consideration of SLN mapping in cases of uterine-confined diseases in the absence of nodal involvement as detected by imaging and on surgical exploration [8]. However, practice variability exists for lymph node assessment in “low risk” disease. Based on our study, the pathological features of low risk disease include **1**) *lack of LUSI*, **2**) *a tumor size of less than 5 CM*, **3**) *lack of histological LVI*, **4**) *lack of CSI*, and **5**) *a depth of invasion of less than 50%*. There are studies that have evaluated some of these features individually [9, 24–26], but in the current study, we have made an attempt to stratify them collectively and emphasize their degree of importance. For example, Frimer et al. and Kim et al. had mainly associated the depth of myometrial invasion with SLN involvement [9, 25]. It should be emphasized that intraoperative pathologic assessment (frozen section) of hysterectomy specimens is essential to assess the extent of tumor involvement beyond the boundaries of corpus uteri [26].

A number of studies have investigated sentinel lymph node mapping and ultrastaging techniques which have shown their use in comparison to more traditional methods such as lymphadenectomy, particularly for low risk disease [27–29]. Omitting lymphadenectomy holds the risk of inadequate staging, while SLN biopsy may be an acceptable alternative to the patients who would not benefit from complete lymphadenectomy [24]. Additionally, Blakely et al. have suggested that incorporation of SLN biopsy during the initial surgery is useful to offset frozen section diagnostic errors [26].

The significance of ITCs is not well understood, and even the significance of micrometastases in the sentinel lymph nodes are not clear to some investigators [30]. On the other hand, some investigators believe that the prognosis of patients with well-differentiated stage 1a carcinomas and ITC or micrometastatic carcinoma in the sentinel lymph nodes is similar to that of patients with no lymph node involvement [31]. Also, ITC does not alter the tumor-staging at the present time [13]. There were only a few cases with ITCs and micrometastatic carcinomas in our series. A larger cohort of patients and a much longer follow up time are needed to assess their clinical significance.

There was no statistically significant difference between the histological grades of the tumors (S7 Table) and SLN involvement, even when grades I and II were combined as low-grade endometrial cancer [19]. Also, there was no correlation between T-MSI and LUSI (S6 Table) also did not yield a significant difference, contrary to the findings in Lynch syndrome [32].

Assessment of the pathological features would lead us to the next important parameter which is tumor staging. In our analysis, we divided pathologic T-stages into “1a” and anything beyond as “>1a”. This division was based on traditional criteria and the current practices as outlined in the algorithms of some institutions for triaging cases to SLN sampling [33]. In our study groups, 18% of patients who did not have SLN involvement (Group-I) had a “>1a” stage as opposed to 70% in Group-II who did have the involvement and parallels the pathological features. The size of the primary tumor is not used for staging of endometrial tumors [13]. However, the investigators at Mayo Clinic have indicated that they had used the tumor size to triage patients to lymphadenectomy and surgical staging during intraoperative consultation [34, 35]. Based on the results of our study, larger tumor size is associated with SLN positivity, supporting the findings of the Mayo Clinic researchers. Currently, only DI and CSI result in changing the T-stages. LUSI has emerged as a prominent feature associated with SLN involvement with 100% sensitivity and negative predictive value as well as a high diagnostic accuracy (87%) as listed in Table 4. It is worth reminding that lower uterine segment and cervical lymphatic drainage occurs to external iliac and common iliac lymph nodes through the parametria. On the other hand, the uterine corpus drains not only to the external iliac but also to interiliac, common iliac, and obturator nodes as well. Uterine fundal drainage occurs consistently through the ovarian lymphatic channels to the infra-renal and para-aortic regions [36]. The findings in this study are based on a small series and warrant further evaluation in the context of a larger prospective cohort. Our study provides further support for incorporation of SLN mapping in all cases of endometrial carcinomas.

Despite the limitations due to a relatively small sample size, the findings in this series as well as in other studies, support SLN resection for endometrial cancers [21, 26, 37, 38]. This study identifies the following pathologic features as being highly associated with SLN involvement: **1**) *lower uterine segment involvement*, **2**) a *tumor size of 5 CM or greater*, **3**) *presence of histological lymphovascular invasion*, **4**) *cervical stromal involvement*, *and* **5**) *a depth of myometrial invasion of ≥50%* while histological grades or microsatellite instability play no significant role. LUSI alone appears to be the most important feature associated with SLN involvement. Although routinely reported as a finding in the pathology reports, LUSI does not change the tumor stage of the cancer per se [13]. The significant correlation of LUSI with SLN positivity, as found in this study, warrants consideration for including LUSI in the staging criteria for endometrial carcinomas, potentially changing the tumor stage to “>1a.” This study systematically stratifies and recaps the importance of the five pathological features that correlate with SLN positivity and collectively may serve as a guide for pathologists and oncologists in their diagnostic and therapeutic planning as well as prognostication assessment. For clinicians, these features may be helpful in deciding which patients would benefit from additional therapy, particularly if no lymph node resection had occurred at the time of operation. Future studies with a statistically powerful sample size are needed to validate our findings.

## Supporting information

S1 Table Summary of the findings in the patients who underwent sentinel lymph node resection for the endometrial lesions. (PDF)

S2 Table Summary of the patients’ median age and pathological findings as well as the tumor stages for all 70 cases classified under the respective diagnoses. (PDF)

S3 Table Summary of the findings in the patients in Group-I with uterine carcinomas who had no lymph node involvement. (PDF)

S4 Table Summary of the features in Groups I & II based on absence or presence of metastasis in the lymph nodes. (PDF)

S5 Table Summary of the findings in the patients with uterine carcinomas who had lymph node involvement. (PDF)

S6 Table Fisher’s Exact test comparisons of tumor microsatellite instability in relation to the sentinel lymph node status and lower uterine segment involvement. (PDF)

S7 Table Fisher’s Exact comparison of the SLNs status versus histological grade. (PDF)

S8 Table Test performance of the five pathological features showing distribution of the true negative, false positive, true positive, and false negative designation of the results. (PDF)
