# Supplementary material for: The uterine pathological features associated with sentinel lymph node metastasis in endometrial carcinomas
Source: PLoS One. 2020 Nov 24;15(11):e0242772. doi: 10.1371/journal.pone.0242772 (PMC7685478; doi:10.1371/journal.pone.0242772)
Supplement: S2 Table — (PDF) [file pone.0242772.s002.pdf]

S2 Table. Summary of the patients' median age, pathological findings as well as the tumor stages for all 70 cases classified under the respective diagnoses.

Total = 70	**CAH**	**ECA**	**NRT**	**SCA**	**ECA + SCA**
n (%)	7 (10%)	54 (77%)	2 (3%)	7 (10%)	61 (87%)
Median Age	57	62	50	72	65
**Neg** Lymph Nodes	7 (100%)	44 (81%)	2 (100%)	6 (86%)	50 (82%)
**Pos** Lymph Nodes	0 (0%)	10 (19%)	0 (0%)	1 (14%)	11 (18%)
Histological Grade I	NA	31 (58%)	NA	0 (0%)	31 (50%)
Histological Grade II	NA	18 (33%)	NA	0 (0%)	18 (30%)
Histological Grade III	NA	5 (9%)	NA	7 (100%)	12 (20%)
LUSI	NA	17 (31%)	NA	2 (29%)	19 (31%)
CSI	NA	6 (11%)	NA	0 (0%)	6 (10%)
LVI	NA	8 (15%)	NA	1 (14%)	9 (15%)
Mean, Tumor Size (CM)	NA	3.34 (± 2.14 SD)	NA	1.71 (± 1.76 SD)	3.15 (± 2.15 SD)
Depth of Invasion, <50%	NA	40 (74%)	NA	6 (86%)	46 (75%)
Depth of Invasion, **≥50%**	NA	14 (26%)	NA	1 (14%)	15 (25%)
T-Stage, 1a	NA	38 (70%)	NA	6 (86%)	44 (72%)
T-Stage, **>1a**	NA	16 (30%)	NA	1 (14%)	17 (28%)
T-MSI	NA	15 (28%)	NA	1 (14%)	16 (26%)

CAH, complex atypical hyperplasia; **ECA**, endometrioid adenocarcinoma; **NRT**, no residual tumor identified; **SCA**, serous carcinoma; **Neg**, negative; **Pos**, positive; **Grade**, FIGO histological grade; **NA**, not applicable; **LUSI**, lower uterine segment involvement; **CSI**, cervical stromal involvement; **LVI**, lymphovascular invasion; **CM**, centimeters; **SD**, standard deviation; **>1a**, includes 1b, 2, 3, and 3a; **T-MSI**, Tumor Microsatellite Instability.
