# Supplementary material for: The uterine pathological features associated with sentinel lymph node metastasis in endometrial carcinomas
Source: PLoS One. 2020 Nov 24;15(11):e0242772. doi: 10.1371/journal.pone.0242772 (PMC7685478; doi:10.1371/journal.pone.0242772)
Supplement: S3 Table — (PDF) [file pone.0242772.s003.pdf]

S3 Table. Summary of the findings in the patients with uterine carcinomas who had no sentinel lymph node involvement.

NO.	Age	OBxDx	BxFG	RDx	RFG	SLNDx	SLN/LN Site	LUSI	CSI	LVI	T-Size	DI-FS	DI-PS	T-Stage	Microsatellite Instability		
															T-MSI	MLH-M	G-MSI
**Group I. Endometrioid carcinoma (ECA) with *negative* lymph node involvement**																	
8	52	CAH	NA	ECA	I	N	CI	NI	NI	NI	0.4	NC	<50%	1a	MSH6-		GMND
9	62	CAH	NA	ECA	I	N	P **(L)**	NI	NI	NI	0.5	NC	<50%	1a			
10	39	CAH	NA	ECA	I	N	P	NI	NI	NI	1.5	NC	<50%	1a			
11	67	CAH	NA	ECA	I	N	P	NI	NI	NI	2.2	<50%	<50%	1a			
12	60	CAH	NA	ECA	I	N	P	NI	NI	NI	2.8	<50%	<50%	1a			
13	65	ECA	I	ECA	I	N	PA, P	NI	NI	NI	0.4	NC	<50%	1a			
14	69	ECA	I	ECA	I	N	CI, P **(L)**	NI	NI	NI	0.7	<50%	<50%	1a			
15	57	ECA	II	ECA	II	N	PA, P	NI	NI	NI	0.9	<50%	<50%	1a			
16	56	ECA	I	ECA	I	N	P	NI	NI	NI	1	NIv	<50%	1a			
17	54	ECA	I	ECA	I	N	O, CI, P	NI	NI	NI	1	<50%	<50%	1a	MSH2-, MSH6-		np
18	62	ECA	I	ECA	II	N	PA, CI, P	NI	NI	NI	1.2	<50%	<50%	1a	PMS2-	np	
19	57	ECA	II	ECA	II	N	P	NI	NI	NI	1.5	<50%	<50%	1a			**GMD**
20	56	ECA	I	ECA	II	N	P	NI	NI	NI	1.5	<50%	<50%	1a			
21	68	ECA	I	ECA	I	N	P	NI	NI	NI	1.5	NF	<50%	1a			
22	74	ECA	II	ECA	I	N	P	NI	NI	NI	1.7	<50%	<50%	1a			
23	61	ECA	III	ECA	III	N	PA, P	NI	NI	NI	1.7	NIv	<50%	1a	MLH1-, PMS2-	**MD**	
24	55	ECA	I	ECA	I	N	PA, P	NI	NI	NI	1.8	<50%	<50%	1a			
25	61	ECA	III	ECA	III	N	PA, P	NI	NI	NI	2	<50%	<50%	1a			
26	62	ECA	I	ECA	I	N	PA, P	NI	NI	NI	2.1	<50%	<50%	1a			
27	58	ECA	I	ECA	I	N	PA, P	NI	NI	NI	2.1	<50%	<50%	1a	MLH1-, PMS2-	**MD**	
28	72	ECA	I	ECA	I	N	P	NI	NI	NI	2.1	**≥50%**	**≥50%**	**1b**			
29	73	ECA	I	ECA	I	N	P **(L)**	NI	NI	NI	2.3	NIv	<50%	1a	MLH1-, PMS2-	**MD**	
30	57	ECA	I	ECA	I	N	P	NI	NI	NI	2.5	<50%	<50%	1a			
31	56	ECA	I	ECA	I	N	PA, P **(L)**	NI	NI	NI	2.5	<50%	<50%	1a			
32	70	ECA	I	ECA	I	N	PA, P	NI	NI	NI	2.5	**≥50%**	**≥50%**	**1b**			
33	67	ECA	I	ECA	I	N	CI, P	NI	NI	NI	3	<50%	<50%	1a			
34	74	ECA	I	ECA	I	N	CI, P **(L)**	NI	NI	NI	3.2	<50%	<50%	1a			
35	66	ECA	II	ECA	II	NES	CI, P	NI	NI	NI	3.5	<50%	<50%	1a	MLH1-, PMS2-	**MD**	

36	85	ECA	I	ECA	I	N	CI, P	NI	NI	NI	3.5	<50%	<50%	1a	MLH1-, PMS2-	MD	
37	71	ECA	I	ECA	I	N	PA, P	NI	NI	NI	3.8	<50%	<50%	1a			
38	77	ECA	I	ECA	I	N	CI, P (L)	NI	NI	NI	4	≥50%	≥50%	1b			
39	60	ECA	III	ECA	III	N	PA, P	NI	NI	Pr	4.2	<50%	<50%	1a			
40	34	ECA	II	ECA	II	N	P	NI	NI	NI	6	≥50%	≥50%	1b			
41	60	ECA	I	ECA	II	N	PA, P	NI	NI	NI	7.2	NF	≥50%	1b			
42	66	ECA	I	ECA	I	N	O, P	NI	NI	NI	1.5	<50%	<50%	1a			
43	60	ECA	I	ECA	I	NES	P	NI	NI	NI	4.1	NF	<50%	1a	MLH1-, PMS2-	MD	
44	33	ECA	I	ECA	I	N	P	Pr	NI	NI	1.8	<50%	<50%	1a			
45	65	ECA	I	ECA	II	N	P	Pr	NI	NI	3.4	≥50%	≥50%	1b			
46	76	ECA	II	ECA	II	N	P	Pr	Pr	NI	5.5	≥50%	≥50%	2	MLH1-, PMS2-	MD	
47	60	ECA	III	ECA	II	N	CI, P	Pr	NI	Pr	5.8	≥50%	≥50%	1b	MLH1-, PMS2-	MD	
48	72	ECA	I	ECA	I	N	PA, P	Pr	Pr	Pr	6.5	≥50%	≥50%	2			
49	73	ECA	II	ECA	II	N	P	Pr	NI	NI	8.3	<50%	<50%	1a	MLH1-, PMS2-	MD	
60	72	ECA, Hx	NA	ECA	II	N	P (L)	Pr	NI	NI	4.5	NF	<50%	1a			
61	53	SCA	III	ECA	III	N	PA, P	NI	NI	NI	3.8	NF	<50%	1a			

Group I. Serous carcinoma (SCA) with *negative* lymph node involvement

64	84	SCA	III	SCA	III	N	P (L)	NI	NI	NI	0.5	NF	<50%	1a	np		
65	79	SCA	III	SCA	III	N	P	NI	NI	NI	0.5	<50%	<50%	1a			
66	69	SCA	III	SCA	III	N	PA, P	NI	NI	NI	0.8	NIv	<50%	1a			
67	65	SCA	III	SCA	III	N	P (L)	NI	NI	NI	1.1	<50%	<50%	1a			
68	72	SCA	III	SCA	III	N	PA, P	NI	NI	NI	1.8	<50%	<50%	1a	MSH6		GMD
69	63	SCA	III	SCA	III	N	PA, P	Pr	NI	NI	1.8	≥50%	<50%	1a			

OBxDx, original biopsy diagnosis; **CAH**, complex atypical hyperplasia; **ECA**, endometrioid adenocarcinoma; **Hx**, by history; **SCA**, serous carcinoma; **BxFG**, biopsy FIGO grade; **NRT**, no residual tumor seen; **RFG**, resection FIGO grade; **NA**, not applicable; **RDx**, resection diagnosis; **SLN**, sentinel lymph node; **LN**, lymph node; **Dx**, diagnosis; **N**, negative; **NES**, negative with endosalpingiosis; **PA**, paraaortic; **P**, pelvic; **CI**, common iliac; **O**, obturator; **(L)**, unilateral, left; **LUSI**, lower uterine segment involvement; **NI**, not identified; **Pr**, present; **LVI**, lympho-vascular involvement; **CSI**, cervical stromal involvement; **T-Size**, tumor greatest dimension in centimeters; **DI-FS**, depth of invasion on frozen sections; **NC**, no carcinoma seen, **NIv**, no myometrial invasion; **NF**, no frozen sections; **DI-PS**, depth of invasion on permanent sections; **T-Stage**, tumor stage; **T-MSI**, tumor microsatellite instability; **np**, not performed; **"-"**, loss of the immunohistochemical reactions; **MLH-M**, MLH methylation; **MD**, MLH methylation detected; **G-MSI**, germline microsatellite instability; **GMD**, MSH6 mutation detected; **GMND**, MSH6 mutation not detected.
Cases with **NC**, **NIv**, and **NF** were considered to have the depth of invasion of <50% intraoperatively.
