# Supplementary material for: The uterine pathological features associated with sentinel lymph node metastasis in endometrial carcinomas
Source: PLoS One. 2020 Nov 24;15(11):e0242772. doi: 10.1371/journal.pone.0242772 (PMC7685478; doi:10.1371/journal.pone.0242772)
Supplement: S4 Table — (PDF) [file pone.0242772.s004.pdf]

S4 Table. Summary of the features in Groups I & II based on absence or presence of tumor in the sentinel lymph nodes.

Total = 60	**Group I**	**Group II**
	**Without LN Involvement**	**With LN Involvement**
n (%)	50 (83%)	10 (17%)
Median Age	64	62
Histological Grade I	28 (56%)	3 (30%)
Histological Grade II	12 (24%)	6 (60%)
Histological Grade III	10 (20%)	1 (10%)
LUSI	8 (16%)	10 (100%)
CSI	2 (4%)	4 (40%)
LVI	3 (6%)	5 (50%)
Tumor Size, Minimum (CM)	0.4	1.8
Tumor Size, Maximum (CM)	8.3	7.5
Tumor Size, Mean (CM)	2.61 (± 1.86 SD)	5.62 (± 1.76 SD)
Depth of Invasion, <50%	41 (82%)	5 (50%)
**Depth of Invasion, ≥50%**	9 (18%)	5 (50%)
T-Stage, 1a	41 (82%)	3 (30%)
**T-Stage, >1a**	9 (18%)	7 (70%)
T-MSI	13 (26%)	3 (30%)

Neg, negative; **Pos**, positive; **LN**, lymph node; **LUSI**, lower uterine segment involvement; **CSI**, cervical stromal involvement; **LVI**, lymphovascular invasion; **CM**, centimeters; **SD**, standard deviation; **>1a**, includes 1b, 2, 3, and 3a; **T-MSI**, Tumor Microsatellite Instability.
