# Supplementary material for: The uterine pathological features associated with sentinel lymph node metastasis in endometrial carcinomas
Source: PLoS One. 2020 Nov 24;15(11):e0242772. doi: 10.1371/journal.pone.0242772 (PMC7685478; doi:10.1371/journal.pone.0242772)
Supplement: S5 Table — (PDF) [file pone.0242772.s005.pdf]

S5 Table. Summary of the findings in the patients with uterine carcinomas who had sentinel lymph node involvement.

NO.	Age	OBxDx	BxFG	RDx	RFG	SLNDx	SLN/LN Site	LUSI	CSI	LVI	T-Size	DI-FS	DI-PS	T-Stage	Microsatellite Instability		
															T-MSI	MLH-M	G-MSI
**Group II. Endometrioid carcinoma (ECA) with *Positive* sentinel lymph node involvement**																	
50	57	ECA	I	ECA	II	**PITC**	**P+**	**Pr**	NI	**Pr**	4.5	<50%	<50%	1a	MLH1-, PMS2-	**MD**	
51	74	ECA	I	ECA	II	**PITC**	**P+**	**Pr**	NI	**Pr**	**5**	<50%	<50%	1a			
52	58	ECA	I	ECA	I	**PITC**	PA, **P+**	**Pr**	NI	NI	**5.5**	**≥50%**	**≥50%**	**1b**			
53	59	ECA	I	ECA	I	**PITC**	PA, **P+**	**Pr**	**Pr**	NI	**7.5**	<50%	<50%	**2**			
54	65	ECA	I	ECA	I	**PM**	PA, **P+**	**Pr**	**Pr**	NI	1.8	NIv	<50%	**2**	MLH1-, PMS2-	**MD**	
55	70	ECA	II	ECA	II	**PM**	**P+**	**Pr**	**Pr**	**Pr**	**6.4**	<50%	**≥50%**	**3**			
56	52	ECA	II	ECA	II	**PM**	**PA+**, **P+ (L)**	**Pr**	**Pr**	**Pr**	**6.5**	<50%	**≥50%**	**3a**	MLH1-, PMS2-	**MD**	
57	79	ECA	I	ECA	III	**PM**	**PA+**, **P+**	**Pr**	NI	**Pr**	**7.5**	**≥50%**	**≥50%**	**3a**			
58	38	ECA	I	ECA	II	**PMi**	**P+**	**Pr**	NI	NI	4.5	<50%	<50%	1a			
59	65	ECA	II	ECA	II	**PMi**	**P+**	**Pr**	NI	NI	**7**	**≥50%**	**≥50%**	**1b**			

OBxDx, original biopsy diagnosis; **CAH**, complex atypical hyperplasia; **ECA**, endometrioid adenocarcinoma; **Hx**, by history; **BxFG**, biopsy FIGO grade; **NRT**, no residual tumor seen; **RFG**, resection FIGO grade; **RDx**, resection diagnosis; **SLN**, sentinel lymph node; **LN**, lymph node; **Dx**, diagnosis; **N**, negative; **NES**, negative with endosalpingiosis; **PITC**, positive isolated tumor cells; **PM**, positive metastatic; **PMi**, positive micrometastasis; **PA**, paraaortic; **P**, pelvic; **"+"**, positive for involvement; **(L)**, unilateral, left; **LUSI**, lower uterine segment involvement; **NI**, not identified; **Pr**, present; **LVI**, lympho-vascular involvement; **CSI**, cervical stromal involvement; **T-Size**, tumor greatest dimension in centimeters; **DI-FS**, depth of invasion on frozen sections; **NIv**, no myometrial invasion; **DI-PS**, depth of invasion on permanent sections; **T-Stage**, tumor stage; **T-MSI**, tumor microsatellite instability; **np**, not performed; **"-"**, loss of the immunohistochemical reactions; **MLH-M**, MLH methylation; **MD**, MLH methylation detected; **G-MSI**, germline microsatellite instability. **NIv**, was considered as <50% depth of invasion intraoperatively.
