# Supplementary material for: The uterine pathological features associated with sentinel lymph node metastasis in endometrial carcinomas
Source: PLoS One. 2020 Nov 24;15(11):e0242772. doi: 10.1371/journal.pone.0242772 (PMC7685478; doi:10.1371/journal.pone.0242772)
Supplement: S6 Table — (PDF) [file pone.0242772.s006.pdf]

S6 Table. Fisher's Exact test comparisons of **tumor MSI** in relation to the sentinel lymph node status as well as LUSI.

S6a Table. T-MSI versus **SLN status**

**Group**	**ECA + SCA**		**ECA**	
	**T-MSI-**	**T-MSI+**	**T-MSI-**	**T-MSI+**
**GI** (Neg SLN)	37 (74%)	13 (26%)	32 (73%)	12 (27%)
**GII** (Pos SLN)	7 (70%)	3 (30%)	7 (70%)	3 (30%)
***P*-Value**	**1**		**1**	

S6b Table. T-MSI versus LUSI

**LUSI**	**ECA + SCA**		**ECA**	
	**T-MSI-**	**T-MSI+**	**T-MSI-**	**T-MSI+**
**LUSI-**	32 (76%)	10 (24%)	28 (76%)	9 (24%)
**LUSI+**	12 (67%)	6 (33%)	11 (65%)	6 (35%)
***P*-Value**	**0.5**		**0.5**	

SLN, sentinel lymph node; **T-MSI**, tumor microsatellite instability; **ECA**, endometrioid adenocarcinoma; **SCA**, serous carcinoma; **Neg**, negative; **Pos**, positive; **"-"**, not detected; **"+",** detected; **LUSI**, lower uterine segment involvement.
