# Supplementary material for: The uterine pathological features associated with sentinel lymph node metastasis in endometrial carcinomas
Source: PLoS One. 2020 Nov 24;15(11):e0242772. doi: 10.1371/journal.pone.0242772 (PMC7685478; doi:10.1371/journal.pone.0242772)
Supplement: S7 Table — (PDF) [file pone.0242772.s007.pdf]

S7 Table. Fisher's Exact comparison of the SLN status versus histological grade (**FIGO**)

**Group**	**ECA + SCA**		**ECA**	
	**Neg SLN**	**Pos SLN**	**Neg SLN**	**Pos SLN**
**Grade I**	28 (56%)	3 (30%)	28 (64%)	3 (30%)
**Grade II**	12 (24%)	6 (60%)	12 (27%)	6 (60%)
**Grade III**	10 (20%)	1 (10%)	4 (9%)	1 (10%)
Grade I	**28**	**3**	**28**	**3**
Grade II	**12**	**6**	**12**	**6**
***P*-Value**	**0.06**		**0.06**	
Grade I	**28**	**3**	**28**	**3**
Grade III	**10**	**1**	**4**	**1**
***P*-Value**	**1**		**0.5**	
Grade II	**12**	**6**	**12**	**6**
Grade III	**10**	**1**	**4**	**1**
***P*-Value**	**0.2**		**1**	
Grades I+II	**40**	**9**	**40**	**9**
Grade III	**10**	**1**	**4**	**1**
***P*-Value**	**0.7**		**1**	

ECA, endometrioid adenocarcinoma; **SCA**, serous carcinoma; **Neg**, negative; **Pos**, positive
SLN, sentinel lymph node
