# Supplementary material for: The uterine pathological features associated with sentinel lymph node metastasis in endometrial carcinomas
Source: PLoS One. 2020 Nov 24;15(11):e0242772. doi: 10.1371/journal.pone.0242772 (PMC7685478; doi:10.1371/journal.pone.0242772)
Supplement: S8 Table — (PDF) [file pone.0242772.s008.pdf]

S8 Table. Test performance (**T-Perf**) of the five pathological features showing distribution of the true negative (**TN**), false positive (**FP**), true positive (**TP**), and false negative (**FN**) designation of the results used for the test measures statistical calculations.

NO.	Groups	RDx	RFG	SLNDx	LN Site	LUSI	LUSI	CSI	CSI	LVI	LVI	T-Size	T-Size	DI	DI
							T-Perf		T-Perf		T-Perf		T-Perf		T-Perf
8	I	ECA	I	N	CI	NI	**TN**	NI	**TN**	NI	**TN**	0.4	**TN**	<50%	**TN**
9	I	ECA	I	N	**P (L)**	NI	**TN**	NI	**TN**	NI	**TN**	0.5	**TN**	<50%	**TN**
10	I	ECA	I	N	P	NI	**TN**	NI	**TN**	NI	**TN**	1.5	**TN**	<50%	**TN**
11	I	ECA	I	N	P	NI	**TN**	NI	**TN**	NI	**TN**	2.2	**TN**	<50%	**TN**
12	I	ECA	I	N	P	NI	**TN**	NI	**TN**	NI	**TN**	2.8	**TN**	<50%	**TN**
13	I	ECA	I	N	PA, P	NI	**TN**	NI	**TN**	NI	**TN**	0.4	**TN**	<50%	**TN**
14	I	ECA	I	N	**CI, P (L)**	NI	**TN**	NI	**TN**	NI	**TN**	0.7	**TN**	<50%	**TN**
15	I	ECA	II	N	PA, P	NI	**TN**	NI	**TN**	NI	**TN**	0.9	**TN**	<50%	**TN**
16	I	ECA	I	N	P	NI	**TN**	NI	**TN**	NI	**TN**	1	**TN**	<50%	**TN**
17	I	ECA	I	N	O, CI, P	NI	**TN**	NI	**TN**	NI	**TN**	1	**TN**	<50%	**TN**
18	I	ECA	II	N	PA, CI, P	NI	**TN**	NI	**TN**	NI	**TN**	1.2	**TN**	<50%	**TN**
19	I	ECA	II	N	P	NI	**TN**	NI	**TN**	NI	**TN**	1.5	**TN**	<50%	**TN**
20	I	ECA	II	N	P	NI	**TN**	NI	**TN**	NI	**TN**	1.5	**TN**	<50%	**TN**
21	I	ECA	I	N	P	NI	**TN**	NI	**TN**	NI	**TN**	1.5	**TN**	<50%	**TN**
22	I	ECA	I	N	P	NI	**TN**	NI	**TN**	NI	**TN**	1.7	**TN**	<50%	**TN**
23	I	ECA	III	N	PA, P	NI	**TN**	NI	**TN**	NI	**TN**	1.7	**TN**	<50%	**TN**
24	I	ECA	I	N	PA, P	NI	**TN**	NI	**TN**	NI	**TN**	1.8	**TN**	<50%	**TN**
25	I	ECA	III	N	PA, P	NI	**TN**	NI	**TN**	NI	**TN**	2	**TN**	<50%	**TN**
26	I	ECA	I	N	PA, P	NI	**TN**	NI	**TN**	NI	**TN**	2.1	**TN**	<50%	**TN**
27	I	ECA	I	N	PA, P	NI	**TN**	NI	**TN**	NI	**TN**	2.1	**TN**	<50%	**TN**
28	I	ECA	I	N	P	NI	**TN**	NI	**TN**	NI	**TN**	2.1	**TN**	**≥50%**	**FP**
29	I	ECA	I	N	**P (L)**	NI	**TN**	NI	**TN**	NI	**TN**	2.3	**TN**	<50%	**TN**

30	I	ECA	I	N	P	NI	**TN**	NI	**TN**	NI	**TN**	2.5	**TN**	<50%	**TN**
31	I	ECA	I	N	**PA, P (L)**	NI	**TN**	NI	**TN**	NI	**TN**	2.5	**TN**	<50%	**TN**
32	I	ECA	I	N	PA, P	NI	**TN**	NI	**TN**	NI	**TN**	2.5	**TN**	**≥50%**	**FP**
33	I	ECA	I	N	CI, P	NI	**TN**	NI	**TN**	NI	**TN**	3	**TN**	<50%	**TN**
34	I	ECA	I	N	**CI, P (L)**	NI	**TN**	NI	**TN**	NI	**TN**	3.2	**TN**	<50%	**TN**
35	I	ECA	II	NES	CI, P	NI	**TN**	NI	**TN**	NI	**TN**	3.5	**TN**	<50%	**TN**
36	I	ECA	I	N	CI, P	NI	**TN**	NI	**TN**	NI	**TN**	3.5	**TN**	<50%	**TN**
37	I	ECA	I	N	PA, P	NI	**TN**	NI	**TN**	NI	**TN**	3.8	**TN**	<50%	**TN**
38	I	ECA	I	N	**CI, P (L)**	NI	**TN**	NI	**TN**	NI	**TN**	4	**TN**	**≥50%**	**FP**
39	I	ECA	III	N	PA, P	NI	**TN**	NI	**TN**	**Pr**	**FP**	4.2	**TN**	<50%	**TN**
40	I	ECA	II	N	P	NI	**TN**	NI	**TN**	NI	**TN**	**6**	**FP**	**≥50%**	**FP**
41	I	ECA	II	N	PA, P	NI	**TN**	NI	**TN**	NI	**TN**	**7.2**	**FP**	**≥50%**	**FP**
42	I	ECA	I	N	O, P	NI	**TN**	NI	**TN**	NI	**TN**	1.5	**TN**	<50%	**TN**
43	I	ECA	I	NES	P	NI	**TN**	NI	**TN**	NI	**TN**	4.1	**TN**	<50%	**TN**
44	I	ECA	I	N	P	**Pr**	**FP**	NI	**TN**	NI	**TN**	1.8	**TN**	<50%	**TN**
45	I	ECA	II	N	P	**Pr**	**FP**	NI	**TN**	NI	**TN**	3.4	**TN**	**≥50%**	**FP**
46	I	ECA	II	N	P	**Pr**	**FP**	**Pr**	**FP**	NI	**TN**	**5.5**	**FP**	**≥50%**	**FP**
47	I	ECA	II	N	CI, P	**Pr**	**FP**	NI	**TN**	**Pr**	**FP**	**5.8**	**FP**	**≥50%**	**FP**
48	I	ECA	I	N	PA, P	**Pr**	**FP**	**Pr**	**FP**	**Pr**	**FP**	**6.5**	**FP**	**≥50%**	**FP**
49	I	ECA	II	N	P	**Pr**	**FP**	NI	**TN**	NI	**TN**	**8.3**	**FP**	<50%	**TN**
50	II	ECA	II	**PITC**	**P+**	**Pr**	**TP**	NI	**FN**	**Pr**	**TP**	4.5	**FN**	<50%	**FN**
51	II	ECA	II	**PITC**	**P+**	**Pr**	**TP**	NI	**FN**	**Pr**	**TP**	**5**	**TP**	<50%	**FN**
52	II	ECA	I	**PITC**	PA, **P+**	**Pr**	**TP**	NI	**FN**	NI	**FN**	**5.5**	**TP**	**≥50%**	**TP**
53	II	ECA	I	**PITC**	PA, **P+**	**Pr**	**TP**	**Pr**	**TP**	NI	**FN**	**7.5**	**TP**	<50%	**FN**
54	II	ECA	I	**PM**	PA, **P+**	**Pr**	**TP**	**Pr**	**TP**	NI	**FN**	1.8	**FN**	<50%	**FN**
55	II	ECA	II	**PM**	**P+**	**Pr**	**TP**	**Pr**	**TP**	**Pr**	**TP**	**6.4**	**TP**	**≥50%**	**TP**
56	II	ECA	II	**PM**	**PA+, P+ (L)**	**Pr**	**TP**	**Pr**	**TP**	**Pr**	**TP**	**6.5**	**TP**	**≥50%**	**TP**

57	II	ECA	III	PM	PA+, P+	Pr	TP	NI	FN	Pr	TP	7.5	TP	≥50%	TP
58	II	ECA	II	PMi	P+	Pr	TP	NI	FN	NI	FN	4.5	FN	<50%	FN
59	II	ECA	II	PMi	P+	Pr	TP	NI	FN	NI	FN	7	TP	≥50%	TP
60	I	ECA	II	N	P (L)	Pr	FP	NI	TN	NI	TN	4.5	TN	<50%	TN
61	I	ECA	III	N	PA, P	NI	TN	NI	TN	NI	TN	3.8	TN	<50%	TN
64	I	SCA	III	N	P (L)	NI	TN	NI	TN	NI	TN	0.5	TN	<50%	TN
65	I	SCA	III	N	P	NI	TN	NI	TN	NI	TN	0.5	TN	<50%	TN
66	I	SCA	III	N	PA, P	NI	TN	NI	TN	NI	TN	0.8	TN	<50%	TN
67	I	SCA	III	N	P (L)	NI	TN	NI	TN	NI	TN	1.1	TN	<50%	TN
68	I	SCA	III	N	PA, P	NI	TN	NI	TN	NI	TN	1.8	TN	<50%	TN
69	I	SCA	III	N	PA, P	Pr	FP	NI	TN	NI	TN	1.8	TN	<50%	TN

CAH, complex atypical hyperplasia; **ECA**, endometrioid adenocarcinoma; **SCA**, serous carcinoma; **NRT**, no residual tumor seen; **RFG**, resection FIGO grade; **NA**, not applicable; **RDx**, resection diagnosis; **SLN**, sentinel lymph node; **LN**, lymph node; **Dx**, sentinel lymph node diagnosis; **N**, negative; **NES**, negative with endosalpingiosis; **PITC**, positive isolated tumor cells; **PM**, positive metastatic; **PMi,** positive micrometastasis; **PA**, paraaortic; **P**, pelvic; **CI**, common iliac; **O**, obturator; **"+"**, positive for metastasis; **(L)**, unilateral, left; **(R)**, unilateral, right; **LUSI**, lower uterine segment involvement; **NI**, not identified; **Pr**, present; **LVI**, lympho-vascular involvement; **CSI**, cervical stromal involvement; **T-Size**, tumor greatest dimension in centimeters; **NC**, no carcinoma seen, **DI**, depth of myometrial invasion; **T-Stage**, tumor stage.
